# Lumbar spine Ewing sarcoma: A report of a rare localization

**DOI:** 10.1016/j.ijscr.2024.110412

**Published:** 2024-10-03

**Authors:** Zyad Al-Frejat, Alia Esper

**Affiliations:** Department of Radiology, Damascus University, Syria

**Keywords:** Case report, Lumbar, Ewing sarcoma, Paediatrics

## Abstract

**Introduction:**

Ewing sarcoma is an aggressive tumor characterized by small round cells and diffuse CD99 positivity seen mainly in Caucasian childhood and adolescent demographics. Ewing sarcoma of spinal origin accounts for approximately 3–9 % of cases most of these cases affecting the sacrum, therefore lumbar lesions are considered quite rare.

**Case report:**

We report a case of lumbar Ewing sarcoma in a 6-year-old female who presented with back pain and neurological symptoms of spinal compression such as lower limb weakness, constipation, and urinary retention. MRI imaging confirmed the presence of a mass located at the level of L4 and L5 vertebrates causing spinal cord compression, The patient underwent local resection of the tumor, and a biopsy was sent for histology and immunophenotyping which affirmed the diagnosis of Ewing sarcoma. The patient was referred to an oncology center for follow-up chemotherapy.

**Clinical discussion:**

Ewing sarcoma is the second most common tumor of childhood and adolescence. However, a tumor arising from the spine is considered rare. It presents with localized symptoms of pain and fever but may cause neural compression symptoms.

Accurate diagnosis relies mainly on radiological scans, histology, and immunohistochemical analysis.

**Conclusion:**

Although Ewing sarcoma of the lumbar spine is rare, it should always be considered among pediatric populations developing localized fever and pain. Early detection is crucial to avoid undesired outcomes.

## Highlights

• Ewing sarcoma is an aggressive small round cell tumor primarily affecting Caucasian children and adolescents, with spinal origin cases being rare, particularly in the lumbar region.
• A 6-year-old female presented with lumbar Ewing sarcoma, exhibiting symptoms like back pain, lower limb weakness, constipation, and urinary retention, confirmed by MRI and histopathology.
• Diagnosis of Ewing sarcoma involves a combination of radiological imaging, histological examination, and immunohistochemical analysis, with CD99 positivity being a key marker.
• local resection of the tumor, followed by referral for chemotherapy, affirming the importance of multimodal treatment approaches in managing Ewing sarcoma.
• Despite its rarity in the lumbar spine, Ewing sarcoma should be considered in pediatric patients presenting with localized pain, fever, and neurological symptoms to ensure early diagnosis and treatment.

## 1 Introduction

Ewing sarcoma is a common malignancy of the bone and soft tissues in pediatric patients [1]. Ewing's Sarcoma (ES) is predominantly seen in the long bones of extremities and commonly involves the femur, pelvis, humerus, tibia, and fibula, but rarely in the spine [2], especially In the non-sacral spine [3], ES is the second most common primary bone malignancy led by osteosarcoma, it is characterized by small round cells with a high nuclear to cytoplasmic ratio, and its differentials include neuroblastoma, alveolar rhabdomyosarcoma and lymphoblastic lymphoma [4].

The most common manifestation of Ewing sarcoma is localized pain accompanied by swelling, but spinal Ewing sarcoma may present with radiculopathy or back pain, and neurological symptoms may be seen due to spinal cord compression, however, these symptoms are delayed therefore metastatic events are likely to be seen [5].

We are reporting this case of lumbar Ewing sarcoma to raise attention to the possibility of this malignancy affecting regions of the spine other than the sacrum, thus ensuring that rare cases of lumbar lesions are detected early and assuring a better prognosis.

## 2 Methods

This case has been reported in line with SCARE criteria [6].

## 3 Case presentation

In this report, we present the case of a six-year-old female who was brought to the hospital by her family due to constipation, urinary retention, and difficulty walking for the past month, the child also suffered from back and lower limb pain three months prior to the current symptoms.

On physical examination, the patient exhibited decreased motor strength in both lower extremities (3/5 in each lower limb), hyperreflexia in the lower extremities, and limb. The remainder of the musculoskeletal and neurological examinations were within normal limits.

Her blood pressure was (100/70 mmHg) and she had a low-grade fever(37.5).

Her laboratory parameters revealed a low hemoglobin level (10.3 g), an extreme erythrocyte sedimentation rate elevation (140 mm/h), a slightly elevated blood phosphorus level (5 mg/dl), and a high CRP level(18).

**Fig. 1:**
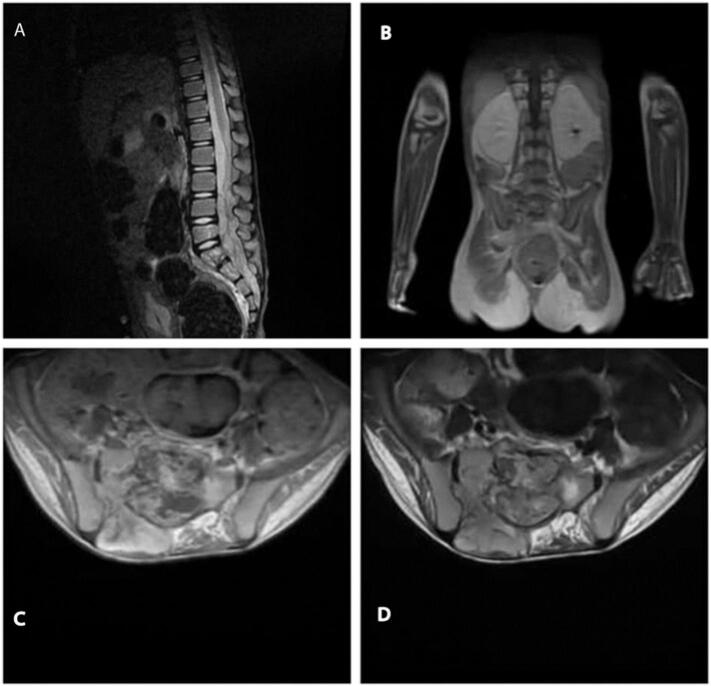
MRI imaging of the spine shows the tumor in (A) the sagittal plane, (B): the coronary plane, (C): T2-weighted transverse plane, and (D): T1-weighted transverse plane.

X-ray for the spine, hips, and knees showed nothing but mild right thoracolumbar scoliosis, there were no observed pathological metabolic changes on the technetium bone scan, and because of the inability to pass urine a voiding cystourethrogram(VCU) was ordered, which showed no pathologic disorders, eventually, the magnetic resonance imaging revealed a soft tissue mass infiltrating the vertebrae. (Fig. 1).

**Fig. 2:**
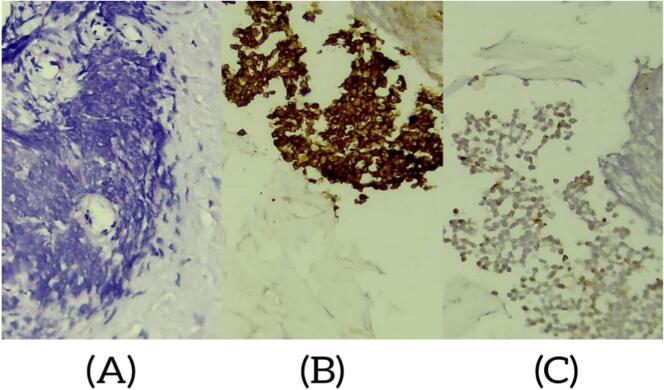
(A). Small round cell tumor (B). Immunostaining with anti CD99 antibody: diffuse membrane staining (C). negative SYN.

No corticosteroids were used and the first treatment was a primary resection of what was possibly a tumor of the L4-L5 laminoectomies, the diagnosis of Ewing's sarcoma was confirmed by the anatomopathological study, that the histopathology showed malignant small round cell tumor that contains connective tissue and bone tissue with necrosis, and immunohistochemistry confirmed that it is a primary central nervous system tumor (PNET/EWING sarcoma) with diffuse positive member staining of tumor cells with CD99 and negative SYN. (Fig. 2).

## 4 Discussion

Ewing sarcoma is an aggressive malignant small round cell tumor [7] first described by James Ewing in 1921 [8], this tumor predominantly affects the metaphysis of long bones with an incidence rate of 80 % of cases [9], vertebral disease is a rare occurrence affecting most commonly the sacrum, our case of lumbar Ewing sarcoma is notably an unusual event [10,11].

Ewing sarcoma has a peak occurrence at a median age of 15 years and a mild male inclination with a 3:2 male-to-female ratio. Caucasians are the most affected ethnicity compared to African and Asian populations, which have a lower occurrence [12].

Symptoms of Ewing sarcoma vary depending on location, primarily local pain is the most encountered symptom in 89.5 % of cases [13], In a substantial number of patients, pain is followed by a palpable soft-tissue mass [14] similarly, our patient suffered from gradually increased pain over 3 months and a mass compressing the spinal canal, causing limb weakness and urinary retention.

A complete medical history should be obtained with care, taking into consideration the associations mentioned between Ewing sarcoma and rare germline mutations mentioned [15].

Although no specific laboratory tests exist for Ewing sarcoma, some studies have correlated high CRP and LDH levels alongside other inflammatory markers with poor prognosis of the tumor [16].

Radiographic imaging is a cornerstone in diagnosing Ewing sarcoma either by CT for osteogenic tumors or by MRI if the tumor arises from soft tissues, both CT and MRI are used for the detection of primary tumor, assessing metastasis and monitoring treatment [17,18].

histology, Immunophenotyping, and the demonstration of specific translocations. Ewing sarcoma expresses CD99 in 90 % of cases and is differentiated from neuroblastoma by the finding of negative synaptophysin [14].

As presented in our case the neurological symptoms arising from the tumor compressing nearby neural structures is an indication for surgery and local resection which can offer the maximum chance for recovery, although surgical options for resection of the tumor vary, the goal is En bloc resection with negative margins. Sacrificing nearby structures and functions by way is a decision left after assessing the total risk of local recurrence and the impact of the outcome versus the functional impairment, noting that the more aggressive the tumor; the more critical it is to obtain safe margins [19].

However postoperative chemical therapy is warranted for micrometastases and helps to develop an idea about the tumor responsiveness to adjuvant therapy [10], The main chemo agents protocol being VACA (vincristine, dactinomycin, cyclophosphamide, and doxorubicin), this protocol is the first line treatment for Ewing sarcoma affecting the spine and can achieve results similar to primary surgery in the fields of preserving neurological function [20].

## 5 Conclusion

Although Ewing sarcoma is the second most common tumor in childhood and adolescence, primary Ewing sarcoma of the non-sacral spine is a Significantly rare aggressive tumor. Chronic back pain accompanied by the development of a mass at the site of pain and possibly neurologic compression symptoms should trigger the suspicion for this diagnosis which is then guided by convenient imaging measures and finally affirmed by histology and immunophenotyping and Treatment must be administered as early as possible for the best prognosis.

## Consent

Written informed consent was obtained from the patient's parents/legal guardian for publication and any accompanying images. A copy of the written consent is available for review by the Editor-in-Chief of this journal on request.

## Ethical approval

This study was approved by the board of the Children's Hospital of Damascus University and by the dean of the faculty of medicine at Damascus University. Ethical approval was not required according to that.

## Funding

No funding was required.

## Author contribution

Zyad Al-Frejat collected the data, participated in the academic writing, and drafted the manuscript.

Alia Esper contributed to the academic writing and extracted the references.

All authors read and approved the final version of the manuscript.

## Guarantor

Dr. Zyad Al-Frejat.

## Research registration number

N/A.

## Declaration of competing interest

The authors declare that there is no conflict of interest.
